# Preparation and Evaluation of Valsartan Liquid Filling Formulations for Soft Gels

**DOI:** 10.1155/2013/418346

**Published:** 2013-01-17

**Authors:** Jyothi Sanaboina, K. M. Maheswari, Seetha Sunkara, Sravanthi Deekonda, Buchi N. Nalluri

**Affiliations:** Department of Pharmaceutics, KVSR Siddhartha College of Pharmaceutical Sciences, Siddhartha Nagar, Andhra Pradesh, Vijayawda 520010, India

## Abstract

The present investigation includes the preparation of liquid filling formulations for soft gels using an antihypertensive drug, valsartan (VAL), in order to improve its dissolution properties and thereby its bioavailability. Formulations were prepared using excipients like polyethylene glycol 400 (PEG 400), propylene glycol (PG), polyvinylpyrrolidone (PVP K-30), antioxidants, ethanol, and purified water. Prepared formulations were evaluated for appearance, pH, drug content percentage, viscosity, stability, and *in vitro* dissolution studies. The compatibility between the drug and excipients in formulations was confirmed by FTIR spectra. The drug contents were in the range of 99.62-99.63 and the viscosity was in the range of 60.9–591.7 cps with all the formulations developed. Formulations containing 10 mg PVP K 30 gave better dissolution properties when compared to formulations without PVP K 30, and a complete drug dissolution was observed within 10 min and followed the first-order release kinetics. Stability studies were conducted for selected formulations (F4–F9) for a period of 6 months at room temperature (*~*30°C/65% RH). From the studies, it can be concluded that VAL liquid filling formulations for soft gels were successfully prepared with *in vitro* dissolution properties superior when compared to VAL itself.

## 1. Introduction

It is estimated that more than 40% of new chemical entities (NCEs) coming out of the current drug discovery process have poor biopharmaceutical properties, such as low aqueous solubility and/or permeability (BCS class II or class IV) [1, 2]. They show extremely low aqueous solubility throughout the physiological pH range, resulting in low and inconsistent bioavailability when administered as solid oral dosage forms. Liquids, in contrast, generally have better bioavailability and one such liquid dosage form is soft gel [2]. The soft gel dosage form offers several advantages over other oral solid dosage forms, such as delivering a liquid matrix designed to solubilize and improve the oral bioavailability of a poorly soluble compound as a unit dose solid dosage form, delivering low and ultralow doses of a compound [3].

VAL is a potent, highly selective and orally administered antihypertensive drug, with poor bioavailability ranging 10–35% because of the poor solubility and dissolution. VAL solubility is low in aqueous fluids, especially in gastric fluids its absorption is dissolution rate limited [4, 5]. The drug is rapidly absorbed after oral administration and median *T*_max_ values of 2.75 and 3 hours have been reported after the oral absorption of tablet and capsule formulations, respectively. The reported absolute bioavailability is 23% for the capsule formulation and 39% for a buffer solution [6]. 

From the literature review, it is clearly evident that most of the works were published with cyclodextrin inclusion complexation [7], solid dispersions [8, 9], self-microemulsifying drug delivery system [10], and other solubilization technologies for improving the solubility, dissolution, bioavailability, and pharmacokinetic properties of VAL. However, Mbah CJ studied the solubility of VAL in solvents like ethyl alcohol and propylene glycol and also in some surfactants [11, 12]. No clear reports were published on the liquid filling formulations for soft gel dosage forms in order to improve the *in vitro *dissolution properties and thereby oral bioavailability of VAL. Hence, the present investigation was aimed at developing oral administrable soft gel (liquid filling) pharmaceutical formulations of VAL with improved dissolution properties.

## 2. Material and Methods

### 2.1. Materials

VAL was supplied by Aurobindo Pharma Ltd., Hyderabad, as a gift sample. PVP K 30 (Sisco Research Laboratories, Mumbai), PEG 400 (Central Drug House, Mumbai), propylene glycol (SD Fine Chemicals, Mumbai), butylated hydroxytoluene (Loba Chemie, Mumbai), sodium metabisulfite (Qualigens Fine Chemicals, Mumbai). All the chemicals and reagents used in the study were of analytical grade.

### 2.2. Preparation of Liquid Filling Formulations

Drug fill solution was prepared by accurately weighing required quantities of VAL along with various excipients as shown in Table 1. Initially VAL was dissolved in a half amount of PEG 400 or PG and other ingredients were added under continuous mixing. The solution was mixed until it becomes clear and finally the volume was adjusted with PEG 400. The prepared formulations were sonicated for 3 minutes in order to remove any entrapped air.

### 2.3. FT-IR Analysis

Samples were analyzed using an ATR-FTIR spectrometer (Bruker, Germany). ATR spectra were measured over the wave number range of 4000–500 cm^−1^ at a resolution of 1.0 cm^−1^. The formulations of all samples were simply placed onto the ATR crystal and each sample spectrum was collected. 

### 2.4. Evaluation Parameters for VAL Liquid Filling Formulations

VAL liquid filling formulations were evaluated for appearance, viscosity, pH, and drug content.

#### 2.4.1. Appearance

Clarity and color change are the most important characteristic features of liquid filling formulations. All developed formulations were evaluated for clarity by visual observation against a black background. 

#### 2.4.2. pH

pH is one of the most important parameter involved in the liquid filling formulations. Soft gel formulation should have a pH range between 2.5 and 7.5 [13]. The developed VAL liquid filling formulations were evaluated for pH by using Elico LI 120 pH meter and estimations were carried out in triplicate.

#### 2.4.3. Drug Content

Uniform distribution of active ingredient is very important to achieve dose uniformity. 10 mg of formulation was taken in a 10 mL volumetric flask and dissolved in 5 mL methanol and the volume was made up with the methanol resulting in 2 mg of VAL per 10 mL solution. 1 mL of the above solution was suitably diluted with pH 6.8 phosphate buffer. Finally drug content was estimated using Elico SL 150 UV-visible spectrophotometer in triplicate.

#### 2.4.4. Rheological Studies

The viscosity was measured using Brookfield DV-II + PRO viscometer. The formulation was taken into the cup of viscometer and measured using spindle CP52 at the rotation of 10–100 rpm. The viscosity measurements were made in triplicate using fresh samples each time.

#### 2.4.5. *In Vitro* Dissolution Studies

*In vitro* dissolution studies were conducted using 1000 mL of pH 6.8 phosphate buffer as a dissolution medium using a USP type II paddle method dissolution apparatus (DISSO 8000, LAB INDIA). A temperature of 37 ± 0.5°C and a rotation speed of 50 and 100 rpm were maintained. Liquid formulations were filled into hard capsule (size 1) and dissolution studies were performed. As the capsule tends to float in the dissolution medium, sinkers were used. A 5 mL sample was withdrawn at predetermined time intervals over a period of 2 hrs and then replaced with the same volume of fresh dissolution medium. The filtered samples were suitably diluted and analyzed at 250 nm using UV-visible Elico SL150 spectrophotometer. Dissolution experiments were conducted in triplicate [14]. 

#### 2.4.6. Stability Studies

Stability testing is performed to ensure that drug products retain their fitness for use until the end of their expiration date. Selected liquid filling formulations (F4–F9) were observed for drug content, clarity, color change, and precipitation if any for a period of 6 months at room temperature (~30°C/65% RH). 

## 3. Results and Discussion

### 3.1. Preparation of Liquid Filling Formulations

Liquid filling formulations were prepared using PEG 400, PG as water miscible solvents either alone or in combination, and water or ethanol as vehicle, with and without PVP K 30 and antioxidants. Prepared formulations were evaluated for further studies. 

### 3.2. FT-IR Analysis

**Figure 1:**
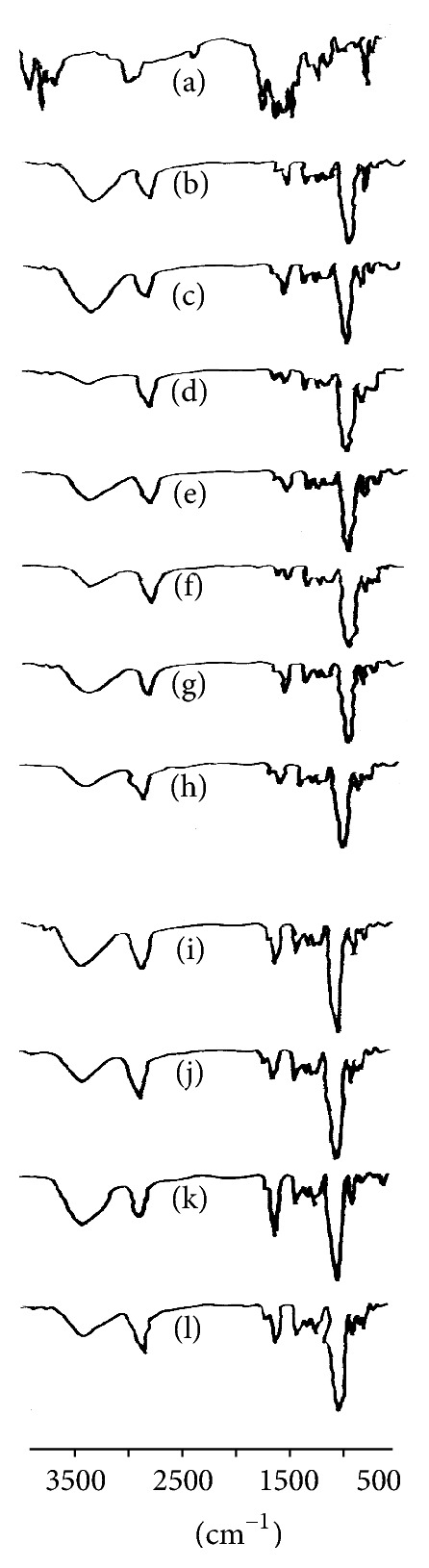
FT-IR spectra of (a)-VAL, (b)-F1, (c)-F2, (d)-F3, (e)-F4, (f)-F5, (g)-F6, (h)-F7, (i)-F8, (j)-F9, (k)-F10, and (l)-F11.

VAL has two characteristic carbonyl absorption bands at 1730 and 1601 cm^−1^ that correspond to carbonyl and amide carbonyl stretching, respectively. The peak at 3563 cm^−1^ indicates the presence of N–H functional group. The band at 2926 cm^−1^ indicates the presence of C–H group stretching vibration. The spectrum reveals the characteristic peaks in the typical range at 1205–1065 cm^−1^ confirms the presence of characteristic tetrazole ring in the VAL. The complex region of 900–600 cm^−1^ indicates skeletal vibration and an aromatic ring in the drug substance. From the overlaid FT-IR spectra as shown in Figure 1, it was confirmed that VAL in liquid state was compatible with different excipients used in the formulation. 

### 3.3. Evaluation Parameters for VAL Liquid Filling Formulations

#### 3.3.1. Appearance

The formulations (F1–F11) were homogeneous and colorless and no precipitation of drug was observed. From Table 2, all the formulations were transparent in appearance.

#### 3.3.2. pH

The pH of the formulations was about 6.0 and was within the limits. From Table 2, the pH of all liquid filling formulations was suitable for further studies.

#### 3.3.3. Drug Content

The drug content was within the acceptable range for all formulations indicating uniform distribution of drug, that is, solubilization of VAL in all the formulations. From Table 2, VAL content was found to be 98.62% to 99.63% with all the formulations prepared. 

#### 3.3.4. Rheological Studies

Viscosity is one of the important parameters which provide vital information during the optimization of the liquid filling formulation for soft gels. In general, the viscosity of liquid filling formulations for soft gels is in the range of 0.222–3000 cps [15]. From Table 2, the viscosity of the formulations (without PVP) F1, F2, F3, F4, and F5 was low when compared to the formulations (with PVP) F6, F7, F8, and F9 based on their consistency. Formulations F1, F2, F3, F4, and F5 were fluid like consistency, whereas formulations F6, F7, F8, and F9 were slightly thick in consistency. The viscosity of formulations F10 and F11 were thicker in consistency and they failed to give viscosity at a higher shear rate (above 10 rpm). The consistency and viscosity of the filling formulations were related to each other because both were dependent on the concentration of PVP K 30. It was clearly evident that the viscosity and consistency of liquid filling formulations were affected by concentrations of PVP K 30 and PEG 400.

#### 3.3.5. *In Vitro* Dissolution Studies

**Figure 2:**
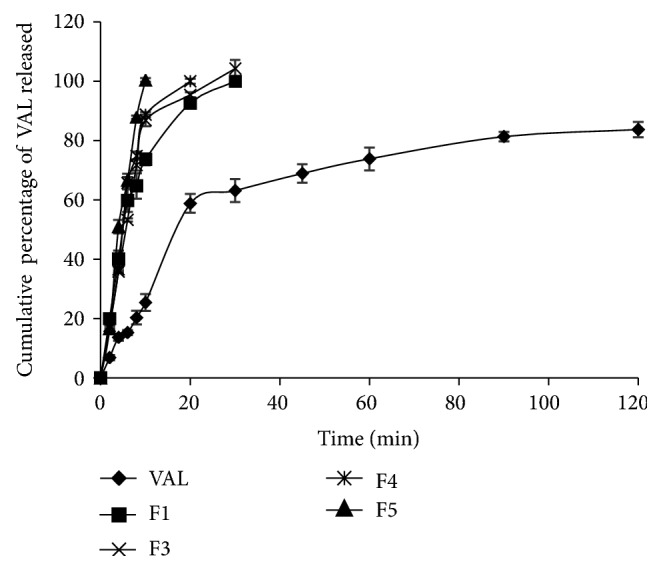
Comparative *in vitro* dissolution profile for VAL and its liquid filling formulations F1, F2, F3, F4, and F5 (*n* = 3).

Totally 11 different liquid filling formulations of VAL were prepared with and without PVP K 30 and antioxidants. The dissolution profiles showed that VAL dissolution was influenced by the solvents containing PVP K 30 rather than antioxidants incorporated in the formulation of the fill liquid. The influence of the solvent system on VAL dissolution was confirmed by comparing the percentage drug release at 10 min (DP_10_) among the investigated formulae. All formulations exceeded 75% of VAL released after 10 min, whereas only 25.4% was dissolved from the pure drug. VAL dissolution from F1 was 73.58 ± 1.96 at the end of 10 min. This was due to the improper solubilization of VAL in PEG 400 and PG. PG was decreased to 10% w/w in F2, resulted in 85.98 ± 0.79 of VAL dissolution at the end of 10 min. This showed that PG in a lower concentration was suitable for dissolution. The effect of PEG 400 on dissolution was studied by preparing F3 without cosolvents and showed 86.75 ± 1.91 of VAL dissolution at the end of 10 min. In the next step, PG as a co solvent at 5% w/w was incorporated in F4 and F5 for increasing the viscosity and thereby reduces the leakage. In F5, water was replaced with ethanol to evaluate the effect on VAL dissolution. F4 and F5 showed 88.98 ± 0.86 and 100.01 ± 0.66 at the end of 10 min and these results indicated that the presence of ethanol in F5 significantly increased the dissolution of VAL. The comparative dissolution profile for formulations F1, F2, F3, F4, and F5 was shown in Figure 2.

**Figure 3:**
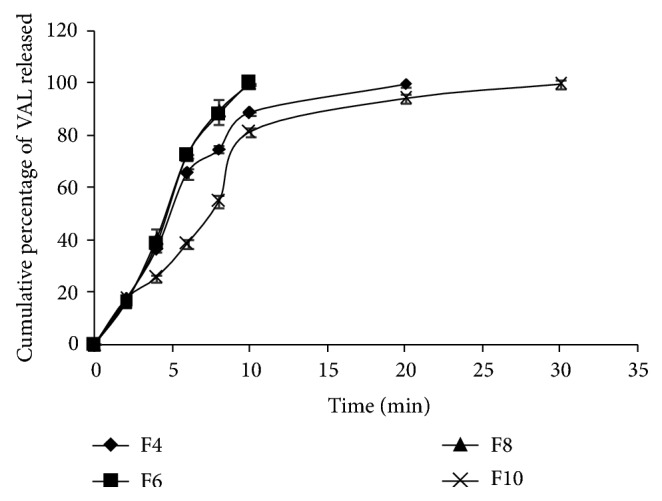
Comparative *in vitro* dissolution profile for liquid filling formulations F4, F6, F8, and F10 (*n* = 3).

**Figure 4:**
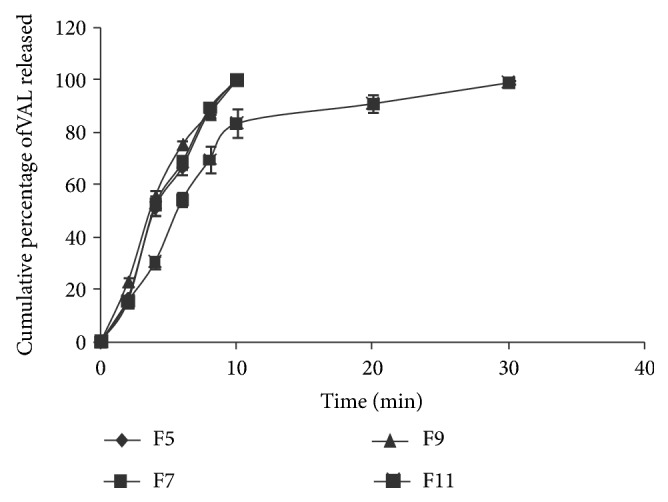
Comparative *in vitro* dissolution profile for liquid filling formulations F5, F7, F9, and F11 (*n* = 3).

Formulations F6 and F7, were prepared by adding PVP K 30 at 5% w/w to evaluate any effect on dissolution of VAL. Addition of PVP K 30 to the formulation F6 when compared to the formulation F4 significantly increased the dissolution properties of VAL and a complete dissolution was observed within 10 min. In the case of F5 and F7, a complete dissolution was observed within 10 min and the addition of PVP K 30 had no effect on the dissolution of VAL to F7. Formulations F8 and F9 were prepared by adding antioxidants (SBS and BHT) to evaluate any effect on dissolution of VAL. The addition of antioxidants in the formulations F8 and F9 did not change/affect dissolution of VAL compared to the F6 and F7 and showed 100.04 ± 0.18 and 100.07 ± 0.02 at the end of 10 min. Formulations F10 and F11 were prepared by adding PVP K 30 at 10% w/w and antioxidants to evaluate any effect on the dissolution of VAL. They showed 81.28 ± 1.82 and 83.81 ± 3.68 of dissolution at the end of 10 min and these values were significantly lower when compared to the values obtained with formulations F8 and F9 and is may be due to the increase in the viscosity and thereby the decrease in the miscibility of liquid filling formulations with the dissolution medium. The dissolution profiles for formulations with and without PVP K 30 were shown in Figures 3 and 4. Overall, formulations F6, F7, F8, and F9 containing 5%, w/w (10 mg/capsule) of PVP K 30 resulted superior dissolution properties of VAL when compared to formulations without PVP K 30 (F1, F2, F3, and F4) and pure VAL. 

#### 3.3.6. Effect of Agitation Speed on Dissolution

Dissolution studies on selected formulations, F4, F8, and F9, were performed at both 50 and 100 rpm in order to evaluate the effect of agitation speed on the dissolution of VAL. The cumulative percent of VAL released at the end of 10 min for formulations F4, F8, and F9 were 88.98 ± 0.86, 100.04 ± 0.18, 100.07 ± 0.02, and 100.36 ± 1.69, 101.60 ± 2.27, and 100.83 ± 1.44, respectively, at 50 and 100 rpm. 

**Figure 5:**
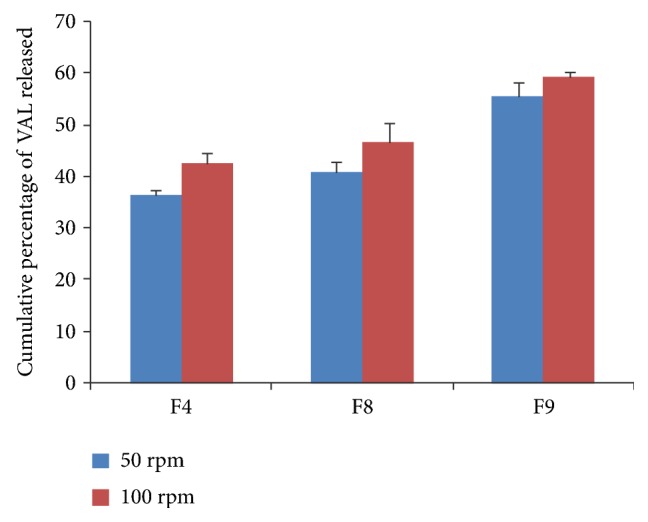
Comparative *in vitro* dissolution profile for F4, F8, and F9 (*n* = 3).

The increase in dissolution was observed for formulation F4 at 100 rpm when compared to 50 rpm. But the formulations F8 and F9 showed a complete dissolution within 10 min irrespective of speed. The comparative dissolution profile was shown in Figure 5. The initial increase in dissolution of formulations at 100 rpm was may be because of the miscibility of liquid filling formulations at higher rpm. Hence, the selection of appropriate rpm was important in the development of soft gel formulations.

#### 3.3.7. Drug Release Kinetics

**Figure 6:**
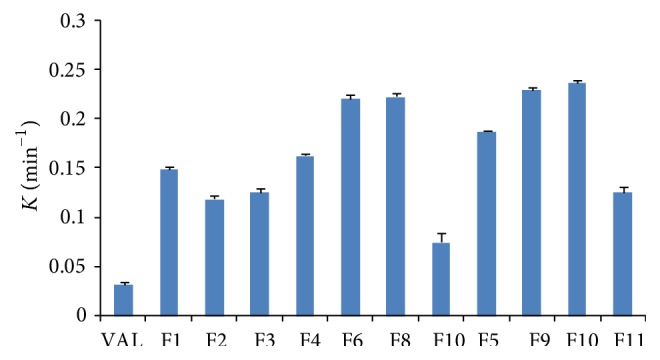
Comparative “*k*” values for VAL and its liquid filling formulations (*n* = 3).

The first-order dissolution rate constant “*k*” value for liquid filling formulations was calculated from dissolution data (0–6 min) by fitting data into a first-order equation. The “*k*” (min^−1^) values for VAL and its liquid filling formulations F1–F11 were 0.032, 0.149, 0.119, 0.126, 0.163, 0.188, 0.221, 0.230, 0.223, 0.237, 0.075, and 0.126, respectively. The comparative profile of “*k*” values for VAL and its liquid filling formulations (F1–F11) was shown in Figure 6.

The 4.65-, 3.71-, 3.93-, 5.09-, and 5.87-fold increases in “*k*” values were observed for formulations F1, F2, F3, F4, and F5 when compared to pure VAL. A 1.15-fold increase in “*k*” value was observed for formulation F5 (PEG/PG/ethanol) when compared to formulation F4 (PEG/PG/water). The “*k*” values were significantly higher for liquid filling formulations containing PVP K 30 when compared to formulations without PVP K 30. The 1.35-and 1.36-fold increases in “*k*” values was observed for formulations F6 and F8 when compared to formulation F4. Similarly, a 1.22 and 1.26 folds increase in “*k*” values was observed for formulations F7 and F9 when compared to formulation F5. Overall, all the liquid filling formulations gave higher release rate constant values when compared to pure VAL. 

#### 3.3.8. Stability Studies

**Figure 7:**
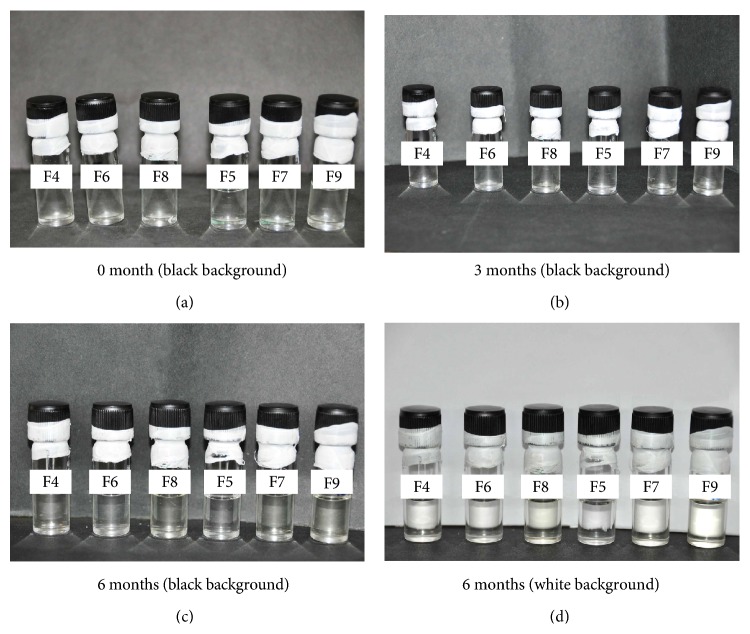
Stability studies data on clarity and color change for VAL liquid filling formulations F4–F9.

**Figure 8:**
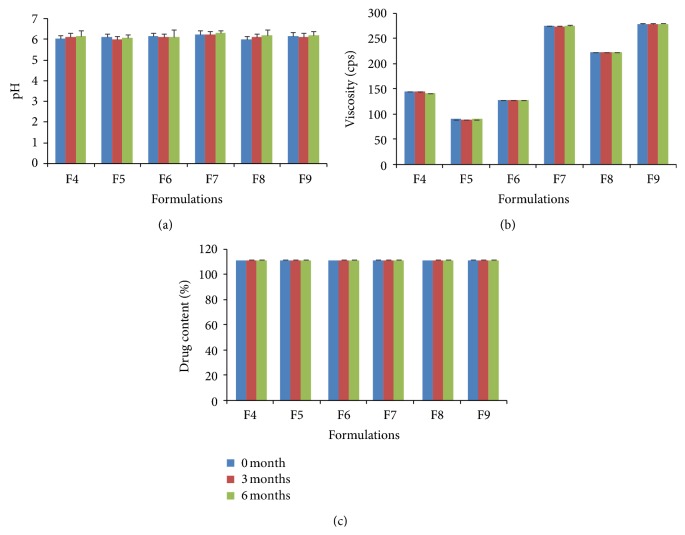
Stability studies data on pH, viscosity, and drug content of VAL liquid filling formualtions F4–F9 (*n* = 3).

The formulations showed no changes in clarity, colour, and precipitation at the end of 3 months. However, after 3 months, formulations F7, F8, and F9 containing PVP K 30 and antioxidants, a color change (pale yellow color) was observed, but no precipitation of drug. The percent VAL contents were also within the limits and the stability data was given in Table 3 and shown in Figures 7 and 8. 

## 4. Conclusion

VAL can be solubilized by the use of a co solvent system (PEG/PG/water or PEG/PG/ethanol) containing a PVP K 30 (5% w/w) in liquid filling formulations and showed improved dissolution properties when compared to the VAL alone in powder form. Liquid filling formulations with PEG/PG/ethanol gave the superior results when compared to formulations containing PEG/PG/water. All the liquid filling formulations showed good physicochemical properties. The formulations were stable up to 6 months without undergoing any degradation. However, after 3 months, the color change (pale yellow) was observed with formulations F7, F8, and F9.

## Figures and Tables

**Table 1:** Liquid filling formulations of VAL.

Ingredients (mg/cap)	F1	F2	F3	F4	F5	F6	F7	F8	F9	F10	F11
Valsartan	40	40	40	40	40	40	40	40	40	40	40
PVP K 30	—	—	—	—	10	10	20	—	10	10	20
PEG 400	100	100	160	130	120	120	110	130	120	120	110
PG	40	20	—	10	10	10	10	10	10	10	10
BHT	—	—	—	—	—	—	—	—	—	1.0	1.0
SBS	—	—	—	—	—	0.1	0.1	—	—	—	—
Water	20	40	—	20	20	20	20	—	—	—	—
Ethanol	—	—	—	—	—	—	—	20	20	20	20

Total weight	200	200	200	200	200	200	200	200	200	200	200

PVP K 30: polyvinylpyrrolidone; PEG 400: polyethylene glycol; PG: propylene glycol; BHT: butylated hydroxy toluene; SBS: sodium meta bisulfite.

**Table 2:** Evaluation parameters for VAL liquid filling formulations (*n* = 3).

Formulations	Appearance	pH (mean ± SD)	Drug content (%) (mean ± SD)	Viscosity (cps) (mean ± SD)
F1	Clear	6.02 ± 0.21	98.63 ± 0.45	62.8 ± 0.10
F2	Clear	5.96 ± 0.15	98.94 ± 0.37	60.9 ± 0.02
F3	Clear	6.13 ± 0.15	99.10 ± 0.42	87.8 ± 0.06
F4	Clear	6.06 ± 0.15	99.26 ± 0.13	145.5 ± 0.10
F5	Clear	6.11 ± 0.17	99.63 ± 0.28	89.6 ± 0.01
F6	Clear	6.00 ± 0.17	99.76 ± 0.12	128.3 ± 0.05
F7	Clear	6.10 ± 0.20	99.56 ± 0.31	275.5 ± 0.01
F8	Clear	6.01 ± 0.14	99.34 ± 0.29	223.7 ± 0.07
F9	Clear	6.15 ± 0.20	99.61 ± 0.33	280.1 ± 0.01
F10	Clear	6.11 ± 0.13	99.17 ± 0.17	591.7 ± 0.03
F11	Clear	6.10 ± 0.17	98.62 ± 0.28	515.5 ± 0.02

**Table 3:** Stability studies for VAL liquid filling formulations (0–6 M) at room temperature.

Formulations	Initial properties	Time points (months)					
	0 month	1	2	3	4	5	6
F4	Homogeneous, colorless, no precipitation	X∗	X	X	X	X	X
F5	Homogeneous, colorless, no precipitation	X	X	X	X	X	X
F6	Homogeneous, colorless, no precipitation	X	X	X	X	X	X
F7	Homogeneous, pale yellow color, no precipitation	X	X	X	X	∗+	+
F8	Homogeneous, pale yellow color, no precipitation	X	X	X	X	+	+
F9	Homogeneous, pale yellow color, no precipitation	X	X	X	X	+	+

∗X—no change ∗+— and pale yellow colour.
